# Exploring Outcomes by Ethnicity in Allogeneic Hematopoietic Cell Transplantation

**DOI:** 10.3390/cancers17040651

**Published:** 2025-02-14

**Authors:** Elizabeth Herrity, Sanjay Singhabahu, Mats Remberger, Tommy Alfaro Moya, Igor Novitzky Basso, Ivan Pasic, Wilson Lam, Arjun D. Law, Auro Viswabandya, Armin Gerbitz, Rajat Kumar, Dennis D. Kim, Jeffrey H. Lipton, Jonas Mattsson, Fotios V. Michelis

**Affiliations:** 1 Hans Messner Allogeneic Blood and Marrow Transplant Program, Princess Margaret Cancer Centre, Toronto, ON M5G 2C4, Canada; elizabeth.herrity@mail.utoronto.ca (E.H.); sanjay.singhabahu@uhn.ca (S.S.); tommy.alfaromoya@uhn.ca (T.A.M.); igor.novitzkybasso@uhn.ca (I.N.B.); ivan.pasic@uhn.ca (I.P.); wilson.lam@uhn.ca (W.L.); arjun.law@uhn.ca (A.D.L.); auro.viswabandya@uhn.ca (A.V.); armin.gerbitz@uhn.ca (A.G.); rajat.kumar@uhn.ca (R.K.); dr.dennis.kim@uhn.ca (D.D.K.); jeff.lipton@uhn.ca (J.H.L.); jonas.mattsson@uhn.ca (J.M.); 2 Clinical Research and Development Unit, Department of Medical Sciences, Uppsala University Hospital, Uppsala University, 752 37 Uppsala, Sweden; mats.remberger@akademiska.se

**Keywords:** allogeneic hematopoietic cell transplantation, race, ethnicity, retrospective, outcomes

## Abstract

Background: Clinical outcome disparities among racial and ethnic groups have been described following allogeneic hematopoietic cell transplantation (HCT). This study investigated the impact of race and ethnicity on HCT outcomes in a multi-ethnic single-center population. Methods: We analyzed outcomes of 709 allogeneic HCT patients, stratified by racial and ethnic groups, who underwent allogeneic HCT between January 2018 and April 2022. Outcomes examined included overall survival (OS), cumulative incidence of relapse (CIR), non-relapse mortality (NRM), and graft-versus-host disease/relapse-free survival (GRFS). Results: No significant differences in OS, CIR, NRM, GRFS, acute GVHD (aGVHD), or chronic GVHD (cGVHD) were observed. Significant differences in age, use of human leukocyte antigen-mismatched donors (HLA-MM), and HCT-CI comorbidity scores ≥ 3 across racial and ethnic groups were observed. Overall mean age was 58 years, with Black patients having the youngest mean age of 43 (range 22–73) and White patients the highest mean age of 59 (range 18–76) (*p* < 0.001). HCT-CI score ≥ 3 was seen in 35.9% of the entire cohort, varying by race and ethnicity: 60.5% in Black, 41.4% in South Asian, 31.5% in White, and 29.0% in East Asian patients (*p* < 0.001). Utilization of HLA-MM donors (including haploidentical) was 29.2% overall, with highest frequencies in Black (65.1%) and East Asian (45%) patients, and lowest in White patients (20.4%) (*p* < 0.001). Conclusions: Statistically significant differences were observed across self-identified racial and ethnic groups regarding age, HCT-CI ≥ 3, and the use of HLA-MM donors. However, post-allogeneic HCT outcomes did not differ significantly by race or ethnicity. Larger prospective trials are warranted to validate our findings.

## Simple Summary

Clinical outcome disparities among racial and ethnic groups have previously been described following allogeneic hematopoietic cell transplantation. The purpose of our study was to investigate the impact of race and ethnicity on allogeneic hematopoietic stem cell transplantation outcomes in a multi-racial and ethnic single-center population in Toronto, Canada. A total of 709 patients who underwent transplant between 2018 and 2022 were analyzed. While no significant differences were observed in overall survival, relapse, non-relapse mortality, or graft-versus-host disease, we did observe differences in sample size, age, disease indication, use of HLA-mismatched donors, CMV reactivation, and comorbidity scores prior to allogeneic hematopoietic stem cell transplant amongst individual groups. Our conclusions are that, within our diverse population, in a health care system fully funded for all residents, no significant differences in post-transplant outcomes were observed. Our findings highlight the need for continued investigation into additional barriers to transplant access and the identification of further support for underserved groups who may benefit from lifesaving transplantation.

## 1. Introduction

Historically, outcomes following allogeneic hematopoietic cell transplantation (HCT) have demonstrated disparities across different self-identified racial and ethnic groups [1,2,3,4,5]. Inequalities in allogeneic HCT have also been observed regarding access to and utilization of HCT [1,2,6,7], as well as in the observed time interval between primary disease diagnosis and HCT [2].

Race is broadly defined as a social construct used to categorize people based on perceived differences in physical appearance, power, and social hierarchy [8]. In contrast, ethnicity as a term is more multidimensional, reflecting shared cultural norms and sociodemographic characteristics [8]. High-income countries, including Canada and the United States, are increasingly interested in analyzing socioeconomic and ethno-cultural data to improve reporting on population groups experiencing disparities in access and health outcomes [8,9,10]. However, racial and other sociodemographic variables are less studied in hematologic malignancies and allogeneic HCT compared to patients with solid tumors [6].

Biological factors such as polymorphisms at human leukocyte antigen (HLA) loci, pharmacogenetic variations, unfavorable disease characteristics, age at diagnosis, comorbid conditions, and transplant-related factors like donor availability and tolerance of conditioning regimens have been hypothesized to contribute to outcome variations in HCT across different racial and ethnic groups [3,4]. However, Mielcarek et al. [2] found that, in HLA matched related and unrelated HCT, Black patients had an unadjusted mortality hazard ratio (HR) of 1.65 compared to White patients, and this increased risk persisted even following adjustments for donor type, pre-transplantation disease risk, donor and patient biological sex, age, and cytomegalovirus (CMV) serological status of recipient and donor, suggesting other factors at play.

More recently in 2023, a retrospective Center for International Blood and Marrow Transplant Research (CIBMTR) analysis of 5473 HCT patients in the United States found, following univariate analysis, disparities in overall survival (OS) among different race and ethnic groups [11]. OS at 5 years was highest in Asian (74%), followed by Hispanic (70%), then non-Hispanic White (65%), and non-Hispanic Black (65%) patients. The multivariate analysis (MVA), however, did not reveal any significant differences between groups for OS, progression free survival (PFS), cumulative incidence of relapse (CIR), or non-relapse related mortality (NRM) [11]. Nevertheless, significant differences were observed between race and ethnic groups regarding age at HCT, performance status, comorbidity index, disease risk index, and transplant-related factors including donor type, graft type, conditioning regimen, graft-versus-host disease (GVHD) prophylaxis, and the year of transplantation [11].

Access to transplant has also been studied by race and ethnicity, utilizing data from the CIBMTR database, highlighting the increase in mismatched unrelated donor (MMUD) and haploidentical donor (HID) as stem cell sources in racially and ethnically diverse patient populations. HID transplants have increased fourfold from 6% in 2013 to 24% in 2020 [12]. Authors describe that while no significant associations have been observed between race and ethnicity in adjusted modeling, the data have supported the existence of access barriers to HCT beyond finding an appropriate donor in diverse racial and ethnic groups [12]. Abraham et al. similarly identified that despite allogeneic HCT being associated with a nearly 80% decrease in the hazard of leukemia death, certain racial and ethnic groups were less likely to undergo HCT despite indications such as secondary acute myeloid leukemia with complex cytogenetics [6].

The present study examined allogeneic HCT outcomes across different self-identified racial and ethnic groups in patients treated at the Princess Margaret Cancer Centre in Toronto, Canada. We also describe patient and transplant-related variables across these groups. The unique context of Canada’s universal healthcare system, combined with a diverse population in our catchment area—nearly 50% of whom are immigrants and over 50% are non-visible minorities—provides a valuable perspective on allogeneic HCT outcomes and access in the era of alternative donor sources and the use of post-transplant cyclophosphamide (PTCy).

## 2. Patients and Methods

### 2.1. Patients

This retrospective analysis included all adult patients who underwent allogeneic HCT at the Princess Margaret Cancer Centre in Toronto, Canada, between January 2018 and April 2022, totaling 709 patients. This study received approval from the Research Ethics Board of the University Health Network.

### 2.2. Variables

Variables collected included patient sex, age at transplant, self-identified race and/or ethnicity, underlying diagnosis, Hematopoietic Cell Transplant-specific Comorbidity Index (HCT-CI) [13,14], donor type, conditioning regimen, GVHD prophylaxis, cytomegalovirus (CMV) serostatus of donor and recipient, acute and chronic GVHD, disease relapse, CMV reactivation, Epstein–Barr virus (EBV) reactivation, number of days of inpatient stay, and survival status at the end of follow-up.

### 2.3. Self-Identified Race and Ethnicity

The electronic medical record and research database combines self-identified race and ethnicity, often capturing one or the other. Based on the multiethnic population within Toronto and the surrounding catchment area, with 46.5% of the total population identifying as immigrants (statcan.gc.ca) serviced by the Princess Margaret Cancer Centre, broad categories were chosen that reflected major groups present. Definitions of groups and further details on the category listed as “Other” are described in Table 1.

### 2.4. Donor Type

The donor type utilized for allogeneic HCT used in the analysis included HLA 10/10 matched related donors (MRD), 10/10 matched unrelated donors (MUD), 9/10 mismatched unrelated donors, and haploidentical (HID) donors. An HLA-MM is defined as any donor source that is not a 10/10 match (e.g., not a complete match in HLA-A, HLA-B, HLA-C, HLA-DRB1, or HLA-DQB1). Our algorithm for donor selection prioritizes, in order of preference, 10/10 HLA MRD, 10/10 HLA MUD, HID, and 9/10 HLA MM unrelated donors.

### 2.5. GVHD Prophylaxis

GVHD prophylaxis regimens vary by donor source and patients’ underlying comorbidities. Prior to HLA-MM HCT, antithymocyte globulin (ATG) is administered at either 2 mg/kg or 4.5 mg/kg (MMUD and HID, respectively). PTCY is given at 50 mg/kg for 2 days, starting 3 days after HCT. Additionally, cyclosporine A (CsA) is administered at 2.5 mg/kg every 12 h, beginning 5 days after transplant. For HLA 10/10 MUD sources, ATG is administered at 2 mg/kg along with PTCy and CsA, while for MRD ATG is eliminated and mycophenolate mofetil (MMF) is added in combination with PTCy and CsA (starting day 1). In patients with underlying cardiac disease (e.g., left ventricular ejection fraction ≤ 45%), PTCy is eliminated in favor of methotrexate (MTX) or MMF.

### 2.6. Antiviral Prophylaxis and Monitoring

Antiviral monitoring begins with weekly assessments of CMV and EBV levels starting 21 days after transplant or at the time of engraftment, whichever occurs first. From March 2020, letermovir (LET) prophylaxis was initiated for all CMV-seropositive patients who received antithymocyte globulin (ATG) as part of their GVHD prophylaxis and was started at day 21 for a total of 100 days. CMV-seronegative recipients and those not receiving ATG were excluded from LET prophylaxis according to institutional guidelines. CMV reactivation is defined as a CMV PCR level greater than 200 IU/mL, while pre-emptive CMV treatment is initiated at 500 IU/mL. EBV reactivation is considered in the presence of any clinical or radiological evidence of post-transplant lymphoproliferative disease (PTLD) or a peripheral blood EBV viral load greater than 1.5 × 10^3^ IU/mL.

### 2.7. Outcome Measures

OS and relapse-free survival (RFS) were measured from the date of HCT until the date of last follow-up or death, or last follow-up or relapse or death, respectively. CIR was calculated from the date of HCT to relapse, while NRM was calculated from the date of HCT to death from other causes in the absence of relapse. Cumulative incidence of aGVHD, cGVHD, CMV, and EBV reactivations were calculated at relevant time points. Additionally, the composite endpoint of GVHD-free relapse-free survival (GFRS), reflecting the time during which patients are alive and free from both relapse and GVHD, was included in the analysis.

### 2.8. Statistical Analysis

The Student’s *t*-test and chi-square test were used to evaluate significant differences in demographic and clinical variables between different self-identified races and ethnicities. Kaplan–Meier and log-rank analyses were used to evaluate OS, RFS, and GFRS. Hazard ratios (HRs) were calculated using Cox proportional hazards regression. CIR, NRM, aGVHD incidence, cGVHD incidence, CMV and EBV reactivations were calculated using competing risk regression. For NRM and CIR, relapse and death in the absence of relapse, respectively, were the competing events. For cumulative incidence of aGVHD, cGVHD, CMV and EBV reactivation, death (within 100 d for aGVHD) was the competing event. For cGVHD, only patients surviving more than 100 days were included in the analysis. A multivariate analysis (MVA) was performed using backwards stepwise selection. Univariate analyses (*p*  <  0.05) identified variables for multivariable stepwise regression, eliminating those with *p*  ≥  0.05. All tests were two-tailed, with significance set at *p*  <  0.05 and no *p*-value adjustments. The statistical analysis was performed using Statistica 13.5 (Tibco Software, Palo Alto, CA, USA) and EZR (freely available software by Y Kanda, Saitama, Japan ver. 1.61) [15].

## 3. Results

### 3.1. Patient Baseline Characteristics

This retrospective study included 709 patients. Patient baseline and transplant characteristics are summarized in Table 2. Five ethnic groups were chosen to conduct the analysis: Black, East Asian, South Asian, White, and Other, of which the defining ancestry and/or region is described in Table 1. Of the 709 patients, the largest represented race and/or ethnicity is White patients at 60.9% (*n* = 432/709), followed by East Asian patients (*n* = 100, 14.1%), South Asian patients (*n* = 70, 9.9%), Black patients (*n* = 43, 6.1%), and Other (n = 64, 9.0%, the group composition of which is described in Table 1). The mean age of the cohort was 58 years (range 18–76) with statistically significant variation among race and ethnicities (*p* < 0.001). Black patients had the lowest mean age at 43 years (range: 22–73), while White patients had the highest mean age of 59 years (range: 18–76). The cohort was 56.4% male and 43.6% female, a trend that was consistent across all self-identified racial and ethnic groups. Notably, the gender gap was narrower among Black patients, with a distribution of 51.2% male and 48.8% female, but the difference was not statistically significant. The variation in diagnosis and/or transplant indication was not statistically significant. However, the Black patient group had the highest percentage of patients transplanted for conditions other than acute leukemia or other myeloid malignancies, with 15 out of 43 patients (34.9%). A total of 4/15 Black patients were transplanted for sickle cell disease (SCD), 8/15 for lymphoma, 1 for chronic lymphocytic leukemia (CLL), and 2 for severe aplastic anemia (SAA). All other races and ethnic groups had roughly 8–12% of patients transplanted for other rare indications. HCT-CI scores equal or greater than 3, typically indicating a higher degree of comorbidity, accounted for 35.9% of the entire cohort. Between groups, Black patients had the highest HCT-CI ≥ 3 in 60.5% of patients (*n* = 26/43), followed by Other patients (42.2%), South Asian patients (41.4%), White patients (31.5%), and, lastly, East Asian patients (29.0%). These discrepancies in HCT-CI ≥3 were statistically significant (*p* < 0.001) according to univariate analysis.

### 3.2. Transplant Characteristics

Transplant-related characteristics are described in Table 2. G-CSF-mobilized peripheral blood stem cells (PBSCs) were used in the majority of patients as a graft source, while bone marrow was used in 33 patients (5% of the entire cohort). Evaluating the donor source identified that 70.8% of the entire cohort received an HLA-fully matched donor, while 29.2% received an HLA-MM donor. Across race and ethnic groups, Black patients most frequently received an HLA-MM HCT at 65.1% (*n* = 28/43) compared to White patients that least often received an HLA-MM HCT 20.4% (*n* = 88/432). Falling in between for HLA-MM HCT frequencies were East Asian (*n* = 45/100, 45%), South Asian (*n* = 27/70, 38.6%), and Other (*n* = 19/64, 29.7%). Donor type by self-identified race and ethnicity is described in further detail in Table 2. HID are utilized in 19.9% of the overall cohort. Black patients use HID most frequently *n* = 23/43 (53.5%), while White patients use them least frequently (*n* = 45/432,10.4%). MUD overall are utilized in 47.7% of patients, with White most frequently utilizing MUD at *n* = 261/432 (60.4%) followed by Other (34.4%), South Asian (32.9%), East Asian (29.0%), and Black (7.0%). MRD, mean utilization was 23.1%, they were most frequently utilized in the Other patient group (35.9%), followed by South Asian patients (28.6%), Black patients (27.9%), East Asian patients (26.0%), and White patients (19.2%). MMUD were infrequently utilized, roughly 9.3% of the entire cohort, with largely equal use across race and ethnic groups. GVHD prophylactic regimens included regimens built with a combination backbone of ATG and PTCy, ATG without use of PTCy, PTCy backbone without use of ATG, and a small percentage of other regimens accounting for less than 2.4% of the entire cohort (*n* = 17/709) (Table 2). GVHD prophylactic regimens utilizing a combination of ATG and PTCy were most prevalent encompassing 70.4% of the entire cohort; by race and ethnic groups, the range fell between 59.4% in the Other group and 74.0% in the East Asian group patients. Dose of ATG was documented, with *n* = 193/612, 31.5% of the total cohort, using a higher dose of 4.5 mg/kg compared to *n* = 491/612, 68.5%, using a lower dose of 2 mg/kg. Black patients utilized higher doses of ATG more frequently (66.7% vs. 33.3%) compared to the other race and ethnic groups, likely because of the increased use of HID in the Black cohort. White patients utilized higher doses of ATG only 25.8% of the time, South Asian patients 33.9%, East Asian patients 37.8%, and Other patients 39.6%. When looking at the broader category of T-cell depletion (TcD), including patients that received either or both ATG and PTCY, 97.6% of patients fell within this category with little variation across race and ethnic groups.

### 3.3. Survival

**Figure 1:**
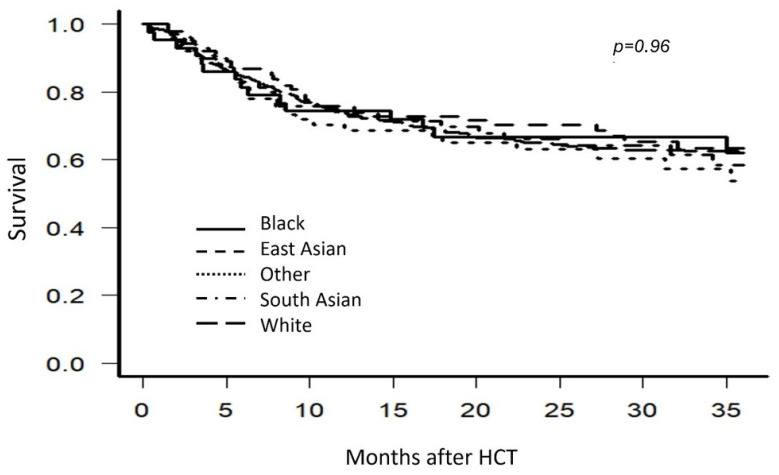
Univariate analysis for OS stratified by self-identified race and ethnicity.

The primary outcome examined was 2-year OS; following univariate analysis, no statistically significant differences were identified between self-identified racial and ethnic groups (*p* = 0.96). East Asian patients had the highest overall survival at 70.3% (range: 60.1–78.3) at two years, followed by Black patients at 66.8% (range: 50.4–78.9), South Asian patients at 66.1% (range: 53.4–76.1), White patients at 65.0% (range: 60.1–69.5), and Other patients at 63.1% (range: 49.7–73.8), as shown in Figure 1.

### 3.4. NRM

**Figure 2:**
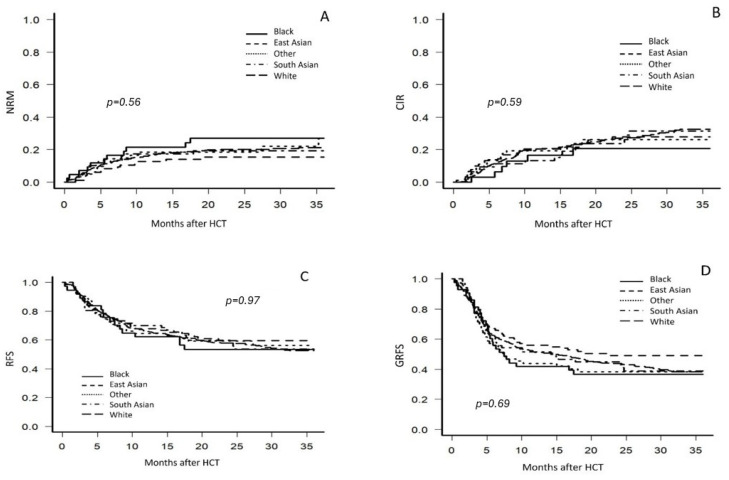
Univariate analysis for non-relapse mortality (NRM) (**A**), cumulative incidence of relapse (CIR) (**B**), relapse free survival (RFS) (**C**), and GVHD-free relapse-free (GRFS) (**D**), stratified by self-identified race and ethnicity.

Following univariate analysis, 2-year NRM did not show statistically significant differences across racial and ethnic groups (*p* = 0.56), as illustrated in Figure 2A. The highest 2-year NRM was observed in Black patients at 25.9% (range: 13.8–39.8), while East Asian patients had the lowest at 14.3% (range: 8.2–22.0). NRM was 19.1% (range: 15.4–23.0) in White patients, 18.8% (range: 10.6–28.9) in South Asian patients, and 17.2% (range: 9.1–27.5) in Other patients.

### 3.5. Relapse

The 2-year CIR showed no statistically significant differences among racial and ethnic groups (*p* = 0.59). Black patients had the lowest CIR at 16.3% (range: 6.5–30.1), while East Asian patients had the highest at 25.3% (range: 16.8–34.7). The CIR for White patients was 23.4% (range: 19.3–27.7), closely followed by Other patients at 23.1% (range: 13.3–34.4) and South Asian patients at 22.4% (range: 12.9–33.6) (Figure 2B).

Differences in 2-year RFS across racial and ethnic groups were not statistically significant (*p* = 0.97). All groups had a mean RFS between 53.5% and 59.5%, with East Asian patients having the highest two-year RFS (range: 28.6–68.6%) (Figure 2C).

### 3.6. GVHD

Univariate analysis showed that day 100 incidence of aGVHD grade II-IV was most prevalent in the Other cohort at 35.9%, with a decreasing trend observed among White (25.9%), South Asian (21.4%), East Asian (17.0%), and Black patients (16.3%), a statistically significant finding (*p* = 0.02). A similar trend was seen in aGVHD grade III–IV, though the incidence ranged between 6–8% across all racial and ethnic groups and was not statistically significant (*p* = 0.56). For cGVHD, Black patients had the highest incidence at 48.7%, followed by South Asian patients at 34.9%, Other patients at 34.5%, East Asian patients at 32.4%, and White patients at 30.0%, though this difference was not statistically significant (*p* = 0.18).

### 3.7. GRFS

No statistically significant differences were found among racial and ethnic groups in the composite endpoint of 2-year GVHD-free, relapse-free survival (GRFS) (*p* = 0.69). Black patients had the lowest two-year GRFS at 36.6% (range: 22.4–50.9), while East Asian patients had the highest GRFS at 49.1% (range: 38.8–58.7) (Figure 2D).

### 3.8. CMV Reactivation

**Figure 3:**
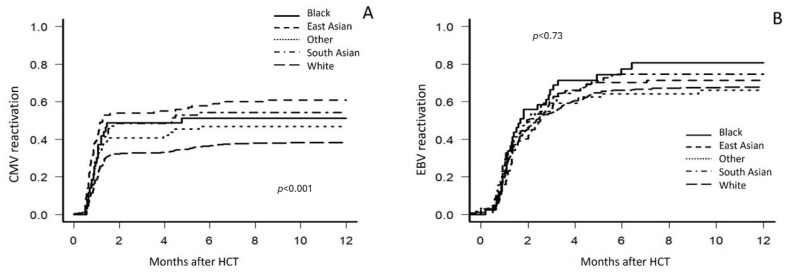
Univariate analysis for cumulative incidence of cytomegalovirus (CMV) and Epstein–Barr virus (EBV) reactivation, stratified by self-identified race and ethnicity. (**A**) refers to the one-year incidence of CMV reactivation. (**B**) refers to the one-year incidence of EBV reactivation.

One-year incidence of CMV reactivation (as previously defined) was most prevalent in East Asian patients at 61.0% (range: 50.6–69.8), followed by 54.3% (range: 41.8–65.2) in South Asians, 51.2% (range: 35.2–65.1) in Black patients, 46.9% (range: 34.2–58.6) in Other, and least common in White patients at 38.3% (range: 33.7–42.9), a statistically significant difference (*p* < 0.001) (Figure 3A).

### 3.9. EBV Reactivation

The one-year incidence of EBV reactivation (any evidence of PTLD or a peripheral blood EBV viral load ≥1.5 × 10^3^ IU/mL) did not show statistically significant differences across racial and ethnic groups (*p* = 0.73). Reactivation was least frequent in the Other group (64.1%), with increasing rates observed in White patients (65.2%), East Asian patients (70.0%), South Asian patients (71.4%), and Black patients (74.4%) (Figure 3B).

### 3.10. Duration of Inpatient Stay

When determining the number of inpatient days on the transplant ward during the initial admission for allogeneic HCT, no significant differences were observed across racial and ethnic groups (*p* = 0.38). Initial admission for transplant days were highest among the Black group (median 35 days) and lowest among the Other group (median 31 days).

When examining the total number of inpatient days, including post-transplant re-admissions, no significant differences were observed across racial and ethnic groups (*p* = 0.20). Total number of inpatient days including readmissions were highest among the Black group (median 42 days) and lowest among the East Asian and Other groups (median 35 days for both).

### 3.11. Multivariate Analysis

The multivariate analysis (MVA) revealed that Black patients had a hazard ratio (HR) of 2.4 (95% CI 1.28 to 4.58, *p* = 0.007) for moderate to severe cGVHD, while the other ethnicity groups had no significant differences. Additionally, the MVA found that Black patients had an HR of 1.5 (95% CI 1.04 to 2.18, *p* = 0.03) for EBV reactivation. Furthermore, East Asian patients had an increased HR of 1.8 for CMV reactivation (95% CI 1.32 to 2.45, *p* = 0.0002), as per the MVA. East Asian patients were less likely to have aGVHD II-IV as per the MVA, with an HR of 0.59 (95% CI 0.39 to 0.89, *p* = 0.01), whereas the Other group was more likely to develop aGVHD II-IV, with an HR of 1.50 (95% CI 1.02 to 2.19, *p* = 0.03).

For all other post-transplant outcomes examined, including grade III-IV aGVHD, MVA demonstrated no impact of the racial and ethnic groups examined.

## 4. Discussion

Addressing health inequities in allogeneic HCT to improve the access and quality of HCT care for vulnerable populations has been recognized as an unmet clinical need in high-income countries [8,9,10]. Health disparities have previously been described in allogeneic HCT access and outcomes across various race and ethnic groups [1,2,3,4,6,7]. Contributing factors included biological and transplant-related parameters, such as inequitable access to matched donors, aggressive disease biology, underlying comorbid conditions, and variable tolerance to conditioning regimens [3,4,11,12].

The present study evaluated for health disparities in allogeneic HCT outcomes in different race and ethnic groups in a diverse cohort of patients serviced by the Princess Margaret Cancer Centre in Toronto, Canada. Re-evaluating disparities previously observed in access and outcomes is warranted due to the increasing use of alternative donor sources, such as haploidentical donors. The use of haploidentical donors has risen considerably over the last few years [16], increasing donor availability for various races and ethnic groups previously underrepresented in registries [12,17]. In relation to this, PTCy as GVHD prophylaxis has decreased the incidence of acute and chronic GVHD, with a tolerable safety profile making its use ever increasing [18,19]. Furthermore, the introduction of letermovir for CMV prophylaxis in seropositive patients to decrease CMV reactivation, a significant cause of transplant related mortality, has become widely adopted [20,21,22,23].

In our study, important outcomes commonly measured post-allogeneic HCT (e.g., OS, NRM, CIR, RFS, GFRS) were not found to be statistically different among various self-identified race and ethnic groups, consistent with findings previously reported that, as HCT supportive care has improved, racial outcome disparities have narrowed [4]. Morishima et al. retrospectively analyzed 26,945 patients receiving MUD HCT between 1988 and 2016 in North America and identified that while favorable HLA characteristics in HCT (e.g., number and location of donor mismatches, patient HLA-B leader genotype, etc.) vary by race, seen most frequently in Japanese and least frequently in Hispanic and Black patients, mortality following HCT continues to decrease. This improvement in survival with respect to time seems most evident in Hispanic and Black patients [4]. Blue et al. retrospectively reviewed CIBMTR data from 5473 patients transplanted in the United States with significant follow-up (up to 5 years); their MVA similarly revealed no significant associations between OS, PFS, relapse, NRM, and race or ethnicity. Their univariate analysis did reveal differences in OS at 5 years, being highest in Asian (74%), followed by Hispanic (70%), non-Hispanic White (65%) and non-Hispanic Black (65%) patients, but no difference in CIR among groups [11].

Among our cohort, statistically significant differences were observed in both donor match and donor type (*p* < 0.001) across racial and ethnic groups. Black patients most frequently received HLA-MM HCT (65.1%), compared to only 20.4% of white patients. Similarly, HID transplants were used in 19.9% of the overall cohort, but most frequently among Black patients (53.5%) compared to White patients (10.4%). Blue et al. reported that while HLA-identical sibling donors were more common, fully matched unrelated donor transplants were most prevalent among non-Hispanic White patients compared to other racial and ethnic minorities [11]. Consistently, White patients in our cohort more often received MUD transplants (60.4%) compared to the overall cohort (47.7%), whereas Black patients had far less frequent MUD transplants (7%). Inequitable access to donor types has been previously described, and increasing the diversity of unrelated donor registries remains a priority. Auletta et al. noted that the use of HID with PTCy for GVHD prophylaxis has been rising, alongside an increasing proportion of HCTs among non-Hispanic White patients [12]. Despite the diversity within our cohort, the sample size for racial and ethnically diverse patients was considerably smaller than that of White patients overall, which may limit our results. However, we did not observe any statistically significant differences in commonly measured post-HCT outcomes.

A significant increase in CMV reactivation in our cohort was observed in East Asian patients at 61.0% (*p* < 0.001), also seen on MVA, with an HR of 1.8 (*p* = 0.0002), compared to other race and ethnic groups in our study. This effect is likely multifactorial. GVHD prophylactic regimens that include ATG and PTCy have been associated with an increased incidence in viral reactivations [24,25,26]. In our observations, the combination of ATG and PTCy was used more frequently in the East Asian group (74%) compared to other racial and ethnic groups, though the ATG dose was generally lower at 2 mg/kg (62.2%). Other factors previously investigated have been the presence or absence of certain HLA alleles in different race and ethnic groups, a complex area of study given the interactions of other transplant variables and clinical events like acute GVHD with CMV reactivation. A previous retrospective analysis from our center by Prem et al. [27] added race as a cofounder in their analysis of the impact of HLA alleles on CMV reactivation in a diverse cohort. No association was found between race and CMV susceptibility; however, the cohort used in that analysis was significantly smaller in size compared to the current one.

Although access to allogeneic HCT was not directly assessed in this study, it should be noted that with the universal societal reimbursed health care system available in Canada, everyone has equal access to HCT on the condition that they are a resident of the province for at least three consecutive months. Nevertheless, our study found significant differences in patient characteristics that were observed across racial and ethnic groups. Specifically, Black patients had a mean age 15 years below the overall cohort mean of 58 years, with South Asian patients also demonstrating a younger mean age of 52 years. Other studies have similarly reported a significantly younger age at the time of HCT among patients of non-White races. For instance, Zhao et al. [1] found that Black patients were younger at diagnosis (54 versus 61 years, *p* < 0.001), and Blue et al. [11] reported that patients from all other racial and ethnic groups, except White, were younger by about a decade at the time of allogeneic HCT (*p* < 0.01). In 2018, SEER database findings did note that the distribution of age at diagnosis differed for all leukemia subtypes across races, with non-Hispanic White patients having the highest age compared to other races [1]. Age, however, at diagnosis in acute myeloid leukemia, a common indication for HCT, is similar among racial groups, and the use of HCT across race and ethnic groups warrants further investigation in this disease group. In our cohort, 4 of the 43 (9.3%) Black patients that underwent transplant had sickle cell disease, possibly influencing the mean age in this group toward a younger trend. Interestingly, Abraham et al. [6] reported that despite a marked prevalence of high-risk molecular features including TP53 mutations, and complex cytogenetic karyotypes reported in Black patients, HCT utilization overall was lower.

Similarly, higher HCT-CI scores, designed to capture organ dysfunction and predict non-relapse mortality risk from allogeneic HCT [13,28], exhibited significant discrepancies across racial and ethnic groups. East Asian patients had the lowest HCT-CI scores, a suggested surrogate for less overall comorbid conditions that would predict better outcomes post HCT, followed by White patients, with South Asian and other groups having higher scores. Notably, Black patients had nearly double the proportion of patients with a high HCT-CI compared to all other groups (60.5% vs. 33.2%, respectively). Abraham et al. [6] similarly found that comorbidity index scores varied among different racial and ethnic groups. However, the hazard ratio of leukemia death was generally lowest in non-Hispanic White patients, a difference that attenuated when proxy variables for structural racism were accounted for. These variables describe differences in environmental exposures, opportunities, and resources available to diverse groups, which can increase their stress-induced biological risk of death [6]. This phenomenon is better described in solid tumor malignancy outcomes, where even in universal payer health care systems such as that used in Canada, systemic and structural racism, marginalization, discrimination, and stigma are believed to compound other factors such as adverse exposures, variations in health behaviors like smoking, poor diet, inactivity, and genetic variants, ultimately negatively impacting outcomes across different societal populations [29,30].

The findings of our study should be interpreted with caution due to several limitations. First, this retrospective analysis involves a relatively small single-center cohort and relies on categorizing individuals into racial or ethnic groups based on self-identification recorded in the electronic medical record, which were subsequently transcribed into our research database. This method does not fully capture the nuanced and potentially significant distinctions between race and ethnicity, which are crucial for health equity research and the application of our findings. There is a notable discrepancy in the number of patients in each cohort (e.g., Black patients compared to White patients) that may introduce bias and limit results. This can be rectified by examining a larger multi-center cohort. Lastly, this study observed significant heterogeneity among the cohorts in terms of transplant age, the presence of increased comorbid conditions, the use of HLA-matched donors, and HCT disease indication across self-identified racial and ethnic groups. These differences could have influenced positively or negatively survival, viral reactivations, and other post-transplant outcomes (e.g., CIR in Black patients could be underestimated given increased prevalence of patients transplanted for SCD [9%] and lymphoma [19%]). Larger registry studies or prospective multicenter trials would provide more clarity with a cohort that is more representative of all racial and ethnic subgroups.

## 5. Conclusions

In conclusion, consistent with other studies investigating barriers to HCT access and outcomes across diverse racial and ethnic groups, it remains crucial to continue examining these barriers, invest in innovative infrastructure solutions, and advocate for enhanced access to high-quality healthcare. This includes minimizing harmful exposures and promoting healthy behaviors to ensure equitable access to allogeneic HCT for all patients with qualifying medical conditions. Prospective studies tracking the use of chemotherapy versus transplant would be particularly valuable in identifying points at which processes fail to transition patients to transplant where it could be of benefit. Multidisciplinary teams are fundamental in facilitating access to support programs, transportation, caregiver services, and in mitigating the stress associated with this complex process.

## Figures and Tables

**Table 1:** Self-identified race and ethnic groups.

Defined Groups	Being From or with Ancestry Linked to the Following Countries and/or Regions Near	
Black	Africa and the Caribbean	
East Asian	East and Southeast Asia including China, the Philippines, Korea, Taiwan, Japan	
Other (*n*, %)	Iran, Iraq, Afghanistan, Lebanon, Egypt	18 (28.1)
	Hispanic	11 (17.2)
	Ashkenazi Jewish	11 (17.2)
	Mixed Race or Ethnicity	6 (9.3)
	Did not report	18 (28.1)
South Asian	India, Pakistan, Sri Lanka, Bangladesh	
White	Europe	

**Table 2:** Patient baseline and transplant characteristics.

Variable							*p*-Value
Race or Ethnicity (*n*, %)	Whole Cohort (*n* = 709)	Black 43 (6.1)	East Asian 100 (14.1)	Other 64 (9.0)	South Asian 70 (9.9)	White 432 (60.9)	
Age (mean, range)	58 (18–76)	43 (22–73)	56 (20–73)	55 (18–73)	52 (19–73)	59 (18–76)	<0.001
Sex (*n*, %)							0.85
Male	400 (56.4)	22 (51.2)	54 (54.0)	34 (53.1)	40 (57.1)	250 (57.9)	
Female	309 (43.6)	21 (48.8)	46 (46.0)	30 (46.9)	30 (42.9)	182 (42.1)	
Diagnosis (*n*, %)							0.25
Acute Leukemia	421 (59.4)	21 (48.8)	62 (62.0)	37 (57.8)	50 (71.4)	251 (58.1)	
CR1	345 (81.9)	15 (71.4)	55 (88.7)	30 (81.1)	35 (70.0)	210 (83.7)	
>/=CR2	76 (18.1)	6 (28.6)	7 (11.3)	7 (18.9)	15 (30.0)	41 (16.3)	
Therapy-related	38 (9.0)	3 (14.3)	3 (4.8)	5 (13.5)	6 (12.0)	21 (8.3)	
Other myeloid ^a^	205 (28.9)	7 (16.3)	30 (30.0)	19 (29.7)	13 (18.6)	251 (58.1)	
Other ^b^	83 (11.7)	15 (34.9)	8 (8.0)	8 (12.5)	7 (10.0)	45 (10.4)	
Donor match (*n*, %)							<0.001
HLA-Matched	502 (70.8)	15 (34.9)	55 (55.0)	45 (70.3)	43 (61.4)	344 (79.6)	
HLA-MM	207 (29.2)	28 (65.1)	45 (45.0)	19 (29.7)	27 (38.6)	88 (20.4)	
Donor Type (*n*,%)							<0.001
Haploidentical	141 (19.9)	23 (53.5)	36 (36.0)	13 (36.0)	24 (34.3)	45 (10.4)	
Mismatched Unrelated	66 (9.3)	5 (11.6)	9 (9.0)	6 (9.4)	3 (4.3)	43 (10.0)	
Matched Unrelated	338 (47.7)	3 (7.0)	29 (29.0)	22 (34.4)	23 (32.9)	261 (60.4)	
Matched Related	164 (23.1)	12 (27.9)	26 (26.0)	23 (35.9)	20 (28.6)	83 (19.2)	
Graft source							0.41
Bone marrow	33 (5)	3 (7)	7 (7)	4 (6)	3 (4)	16 (4)	
TNC ×10^8^ Mean (range) median	2.69 (1.14–6.96) 2.49	2.11(1.22–2.7) 2.41	2.83 (1.14–5.2) 2.32	3.91(1.32–6.96) 3.69	2.17 (1.6–2.88) 2.05	2.5 (1.2–4.2) 2.6	
PBSC	676 (95)	40 (93)	93 (93)	60 (94)	67 (96)	416 (96)	
CD34 ×10^6^ Mean (range) median	7.26 (0.25–25.19) 7.35	7.71(2.49–15.93) 7.54	7.22 (2–25.19) 7.43	7.2 (1.7–18.4) 7.3	7.56 (2.96–17.09) 7.11	7.17 (0.25–20.13) 7.00	
Fresh or Frozen Graft							<0.001
Fresh	531 (74.9)	23 (53.4)	64 (64)	47 (73.4)	53 (75.7)	344 (79.2)	
Frozen	178 (25.1)	20 (46.6)	36 (36)	17 (26.6)	17 (24.3)	88 (20.8)	
GVHD prophylaxis (*n*, %)							0.15
PTCy +/− CNI, MMF or MTX	80 (11.3)	6 (14.0)	17 (17.0)	8 (12.5)	13 (18.6)	36 (8.3)	
ATG +/− CNI, MMF or MTX	113 (15.9)	4 (9.3)	8 (8.0)	15 (23.4)	11 (15.7)	75 (17.3)	
ATG, PTCY +/− CNI, MMF, MTX	499 (70.4)	29 (67.4)	74 (74.0)	38 (59.4)	45 (64.3)	313 (72.5)	
Other	17 (2.4)	4 (9.3)	1 (1.0)	3 (4.7)	1 (1.4)	8 (1.9)	
ATG Dose							<0.001
4.5 mg/kg	193 (31.5)	22 (66.7)	31 (37.8)	21 (39.6)	19 (33.9)	100 (25.8)	
2 mg/kg	419 (68.5)	11 (33.3)	51 (62.2)	32 (60.4)	37 (66.1)	288 (74.2)	
TcD	692 (97.6)	39 (90.7)	99 (99.0)	61 (95.3)	69 (98.6)	424 (98.1)	0.02
HCT-CI ≥ 3 (*n*, %)	247 (35.9)	26 (60.5)	29 (29.0)	27 (42.2)	29 (41.4)	136 (31.5)	<0.001
CMV status (donor negative, recipient negative) (*n*, %)	110 (15.5)	2 (4.7)	4 (4.0)	10 (15.6)	3 (4.3)	91 (21.1)	<0.001

Abbreviations: ATG (antithymocyte globulin); CMV (cytomegalovirus); CNI (calcineurin inhibitor); CR (complete remission); GVHD (graft-versus-host disease); HCT-CI (Hematopoietic Cell Transplantation- specific Comorbidity Index); HLA-MM (human leukocyte antigen—mismatch); kg (kilogram); mg (milligram); MMF (mycophenolate mofetil); MTX (methotrexate); PBSC (peripheral blood stem cell); PTCY (post-transplant cyclophosphamide); TcD (T-cell depletion); TNC (total nucleated cell dose) ^a^ Myeloid malignancies other than acute myeloid leukemia (AML); ^b^ All other transplant indications.

## Data Availability

The datasets presented in this article are not readily available because of our privacy policy. Requests to access the datasets should be directed to Fotios V. Michelis.
